# Controlling the flake size of bifunctional 2D WSe_2_ nanosheets as flexible binders and supercapacitor materials†

**DOI:** 10.1039/d0na00592d

**Published:** 2020-09-30

**Authors:** Pawin Iamprasertkun, Wisit Hirunpinyopas, Varisara Deerattrakul, Montree Sawangphruk, Chakrit Nualchimplee

**Affiliations:** Department of Applied Physics, Faculty of Sciences and Liberal Arts, Rajamangala University of Technology Isan Nakhon Ratchasima 30000 Thailand pawin.ia@rmuti.ac.th; Department of Chemical and Biomolecular Engineering, School of Energy Science and Engineering, Centre of Excellence for Energy Storage Technology (CEST), Vidyasirimedhi Institute of Science and Technology Rayong 21210 Thailand; Department of Chemistry, Faculty of Science, Kasetsart University Bangkok 10900 Thailand; Department of Chemical Engineering, Faculty of Engineering, Kasetsart University Bangkok 10900 Thailand

## Abstract

A new approach using graphene as a conductive binder in electrical supercapacitors has recently been proposed. Graphene shows outstanding properties as a conductive binder, and can be used to replace conductive, additive, and polymer binders. However, graphene follows an EDLC behaviour, which may limit its electrochemical performance. In the process described in this work, we introduced WSe_2_ nanoflakes as a new approach to using pseudocapacitive materials as binders. The WSe_2_ nanoflakes were produced through liquid phase exfoliation of bulk WSe_2_, and the flake size was finely selected using a controlled centrifugation speed. The physical and electrochemical properties of the exfoliated WSe_2_ flakes were analysed; it was found that the smallest flakes (an average flake size of 106 nm) showed outstanding electrochemical properties, expanding our understanding of transition metal dichalcogenide (TMD) materials, and we were able to demonstrate the applicability of using WSe_2_ as a binder in supercapacitor electrodes. We also successfully replaced conductive additives and polymer binders with WSe_2_. The overall performance was improved: capacitance was enhanced by 35%, charge transfer resistance reduced by 73%, and self-discharge potential improved by 9%. This study provides an alternative application of using TMD materials as pseudo capacitive binders, which should lead to the continued development of energy storage technology.

## Abstract 2

WSe_2_ as a multifunctional conductive binder and pseudocapacitive material has been demonstrated. WSe_2_ can be used to replace conductive additive, and polymer binders. The smallest WSe_2_ flakes displayed outstanding electrochemical properties.

## Graphical abstract

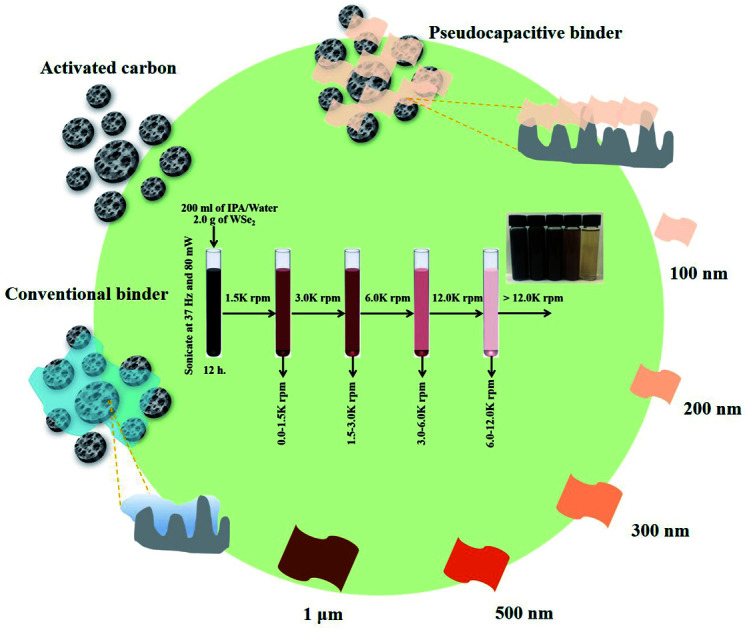

## Introduction

Flexible and freestanding electrodes have been widely applied in energy storage devices (especially supercapacitors) in a process dating back to several decades.^1–3^ The electrochemical performance of energy storage devices is well known, and depends on the physical and electrochemical properties of the electrodes. In the traditional view of the electrodes, the main components are: (1) active materials (∼80%), (2) conductive additives (∼10%), and (3) polymer binders (∼10%).^4^ The development of active materials is central in this context, but the remaining 20%, comprising conductive additives and polymer binders, also play a crucial role in the performance of the device.

The role of a polymer binder such as polyvinylidene fluoride (PVDF) or polytetrafluoroethylene (PTFE) is to bind the materials with the current collector. However, these binders are electrical insulators made from non-capacitive materials, which suppress the overall device performance through poor capacitance and high electrical resistance.^5^ This means that a conductive additive such as carbon black must be used to minimise interfacial resistance.^6^ The polymer binder also blocks some of the pores in the active materials, reducing the overall surface area.^7^ This is a critical issue in the development of large scale energy storage devices.

A new approach to address this issue has been proposed recently by the Bin Xu and Yury Gogotsi,^7,8^ which used reduced graphene oxide (rGO) as a multi-functional conductive binder for fabricating flexible and free-standing carbonaceous electrodes. This strategy has been further expanded to the manufacture of batteries^9–11^ and hybrid capacitor electrodes^12^ using other two-dimensional (2D) materials such as MXene.^13^

However, graphene stores the charge through a build-up in the electrical double layer, which can limit the energy density of the energy storage.^4,14,15^ There are many 2D materials apart from graphene that can be used, such as transition metal dichalcogenides (TMDs, also called MX_2_),^16^ which provide outstanding electrochemical properties in terms of energy storage.^17^ This is due to the charge storage mechanism of these materials based on both faradaic and non-faradaic processes.^18^ This means that we have the opportunity to replace the graphene conductive binders with TMD materials in this context.

Herein, we have introduced WSe_2_ nanoflakes as alternative binder materials to produce a new type of electrode based on the concepts of pseudocapacitive binder. In this experiment, the WSe_2_ nanoflakes were produced through the liquid phase exfoliation of bulk WSe_2_. The flake dimensions were finely tuned by controlling the centrifuge speed, as exfoliated WSe_2_ is produced in different sizes at different revolution speeds. To the best of our knowledge, there have been no prior studies on electrochemical properties based on size dependent WSe_2_ flakes and no attempts have been made to use these materials as pseudo capacitive binders. This work helps the development of electrode manufacturing and is designed to improve high performance energy storage devices.

## Experimental

### Preparation of size selected flakes of WSe_2_

**Fig. 1:**
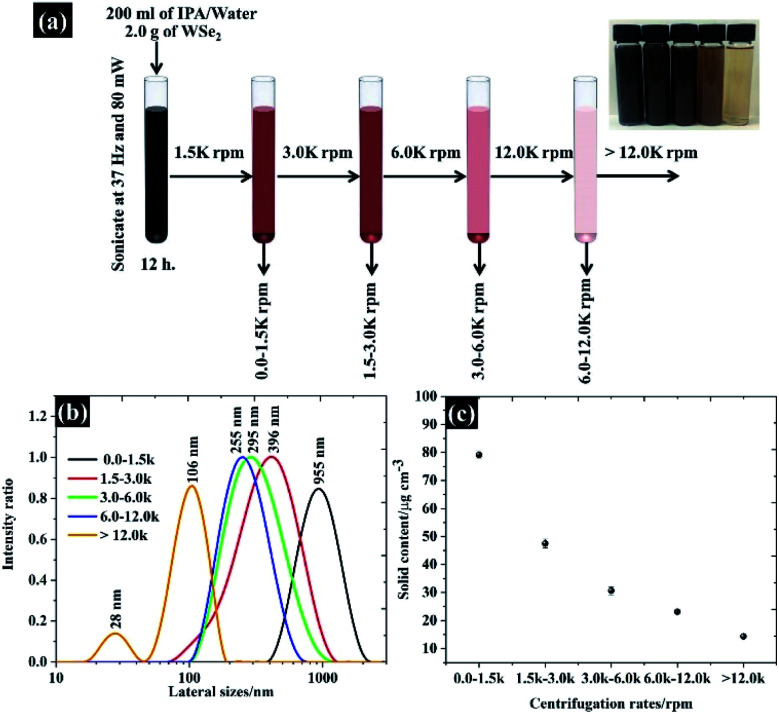
(a) Schematic showing the size selection of WSe_2_ using different centrifugation speeds, (b) the flake size distribution characterised by the dynamic light scattering (DLS) method, and (c) the amount of the solid content obtained from the suspension.

Size selected WSe_2_ nanosheets were prepared through liquid phase exfoliation of bulk WSe_2_ following centrifugation as used in previous procedures.^19–21^ Firstly, 2.0 g of bulk WSe_2_ powder (99%, Sigma-Aldrich) was added into a 200 ml mixture of 1 : 1 v/v water : *n*-propanol (IPA). The suspension was then sonicated at 37 kHz using a power of 80 mW for 12 h. Secondly, the flake size of the exfoliated WSe_2_ was then finely selected by the centrifugal processes as seen in Fig. 1a. The exfoliated WSe_2_ was first centrifuged at 1500 rpm (3139 g) for 30 min. It was necessary to repeat this centrifugal process twice to make sure that all bulk WSe_2_ was removed. The suspension, containing various sizes of WSe_2_ flakes, was detached from the sediment by removing the WSe_2_ supernatant from the top.

According to Coleman *et al.*, the flake sizes of TMD materials diminish with increasing centrifugation speeds;^22^ we therefore controlled the size of WSe_2_ flakes by centrifuging the supernatant at 3000, 6000, and 12 000 rpm. The size controlled WSe_2_ flakes were collected from the sediment after each step (see Fig. 1a). The flake size distribution from the collected sediment is shown in Fig. 1b. Overall, it can be clearly seen that the major population of the flake size was found to be 106, 255, 205, 396, and 955 nm. The exfoliation yield from this technique is shown in Fig. 1c.

### Preparation of the free-standing WSe_2_ electrode

The exfoliated WSe_2_ suspensions were filtered through PVDF membranes (0.1 μm pore diameter, supplied by Millipore Sigma, United States) *via* a syringe pump using an injection rate of 5 ml h^−1^. The prepared electrode was then dried at 50 °C for 24 h. The mass of the active materials was controlled at 0.27 ± 0.02 mg.

### Preparation of the carbon quasi-reference electrode

The quasi-reference electrode was prepared using activated carbon (YEC-8A, with the particle size about 10 μm, surface area ≥ 2100 m^2^ g^−1^, moisture < 5%, ash 0.26%, iron content < 0.005%, purchased from Fuzhou Yihuan Carbon, P. R. China) according to previous experiments.^19,23–26^ Firstly, the activated carbon was dispersed in IPA at a ratio of 0.05 mg ml^−1^. The dispersion was then exposed to ultra-sonication for 1 h. Following this, 20 ml of the prepared activated carbon dispersion was then filtered through PVDF membranes, giving a resultant mass of activated carbon of 1 mg per electrode. The electrochemical performance and stability of the activated carbon quasi-reference electrode were studied by Lee *et al.*, who found that the neutral aqueous electrolyte provided a low level of a potential drift (∼1 mV per day).^26^

### Preparation of the activated carbon electrode using WSe_2_ binder

Overall, 10% by weight of WSe_2_ nanoflakes was added to the active materials for binding the chosen material particles together. Herein, 0.9 mg of activated carbon was added to 10 ml of the prepared WSe_2_ dispersion (flake size of 106 nm, the amount of solid content is about 0.014 mg ml^−1^). The dispersion was then sonicated for 1 h to obtain a homogeneous suspension. Finally, the prepared suspension was filtered through PVDF membranes using a syringe pump with the same injection rate of 5 ml h^−1^. The prepared electrode was then again dried at 50 °C for 24 h. Note, the total mass of the active materials was controlled at about 1.00 ± 0.04 mg.

### Electrochemical evaluation

The coin cell was assembled using a WSe_2_ electrode as the working (positive) electrode, and an activated carbon electrode as the counter/reference (negative) electrode. The electrodes were stacked back to back, and subsequently filled with 0.5 M KCl as an electrolyte. The electrochemical properties of the size dependent WSe_2_ flakes were investigated using various electrochemical techniques in the two-electrode configuration (coin cell CR-2032) using a potentiostat (PGSTAT302N, Metrohm Autolab, Netherlands), running Nova software version 1.11. CV was performed from 0.0 to −0.6 V *vs.* carbon using a scan rate between 10 and 100 mV s^−1^. The galvanostatic charge/discharge (GCD) measurements were taken at 0.5 A g^−1^. Electrochemical impedance spectroscopy (EIS) was applied using a 10 mV perturbation at 0.0 V from 0.1 Hz to 100 kHz.

### Capacitance calculation

The capacitance of WSe_2_ and the prepared activated carbon electrode is based on a single electrode. The capacitance of the electrode was calculated through the integral product of CV using eqn (1).^4^1
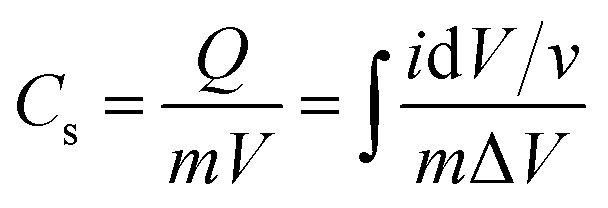
where *m* is the total mass of the electrode (including mass of a binder), *v* is the scan rate, and Δ*V* is the window potential.

### Characterisation of WSe_2_

WSe_2_ dispersions from the different centrifugal speeds were diluted with *n*-propanol to a concentration of 5 μg ml^−1^ (total volume 10 ml). The flake sizes were then measured using the dynamic light scattering (DLS) method, and the size distribution was shown as a *Z*-average figure (average particle diameter). The prepared materials were characterised further by SEM (FEI/Philips XL30 E-SEM, Quanta 650), using an accelerating voltage of 15 kV under high vacuum conditions. To further study the dimension of prepared WSe_2_, the TEM images were obtained from the JEM-ARM200F using an accelerating voltage of 200 kV. Note that the dispersion was drop coated on a copper grid (lacey carbon), and the prepared samples were then cleaned with an ion cleaner (EC-520001C, JEOL) before TEM measurements. The Raman spectra were obtained from a Renishaw inVia Raman microscope using a 532 nm (an excitation energy of 2.33 eV) laser at a laser power of 1 mW. X-ray diffraction (XRD) was performed using a PANalytical X'Pert X-ray diffractometer using Cu-Kα as a radiation source at a wavelength of 0.154 nm, operating at 40 kV and 30 mA. X-ray photo electron spectroscopy (XPS) was performed using a Kratos Axis Ultra DLD spectrometer using the Al Kα as an X-ray source (1486.6 eV). Images of the water contact angle (WCA) were recorded using a Theta Optical Tensiometer (Biolin Scientific, Sweden, running OneAttension software) at a rate of 20 fps. The images were analysed using Young's equation.^27^ The *γ*_sv_, *γ*_sl_, and *γ*_lv_ are solid–vapour, solid–liquid, and liquid–vapour tension respectively. Note that the experiment was carried out in a high humidity chamber to prevent the evaporation of water, and the droplet volume was fixed at 1.0 μl.

2 *γ*_sv_ − *γ*_sl_ − *γ*_lv_ cos *θ* = 0

The electronic conductivities of the prepared electrodes were calculated from *σ* = *L*/*RA* where *R* was measured using the 2 probe method, and *L* and *A* are the thickness and the exposed area of the prepared electrodes.

## Results and discussion

**Fig. 2:**
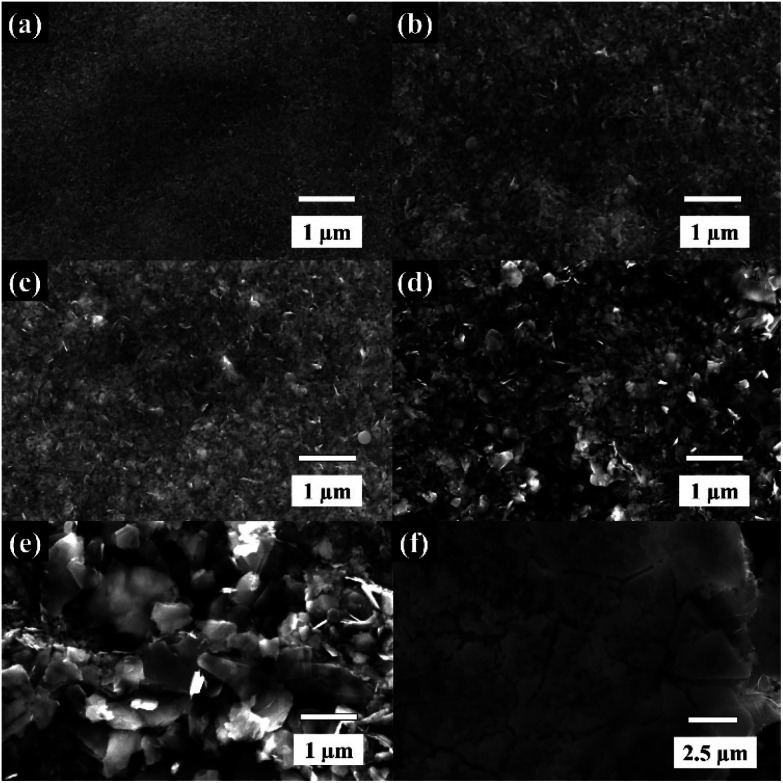
The SEM images showing the size of WSe_2_ flakes at different centrifugal rates (a) ≥12 000 rpm-106 nm, (b) 6000 to 12 000 rpm-255 nm, (c) 3000 to 6000 rpm-295 nm, (d) 1500 to 3000 rpm-395 nm, (e) ≤1500 rpm-955 nm, and (f) bulk material.

**Fig. 3:**
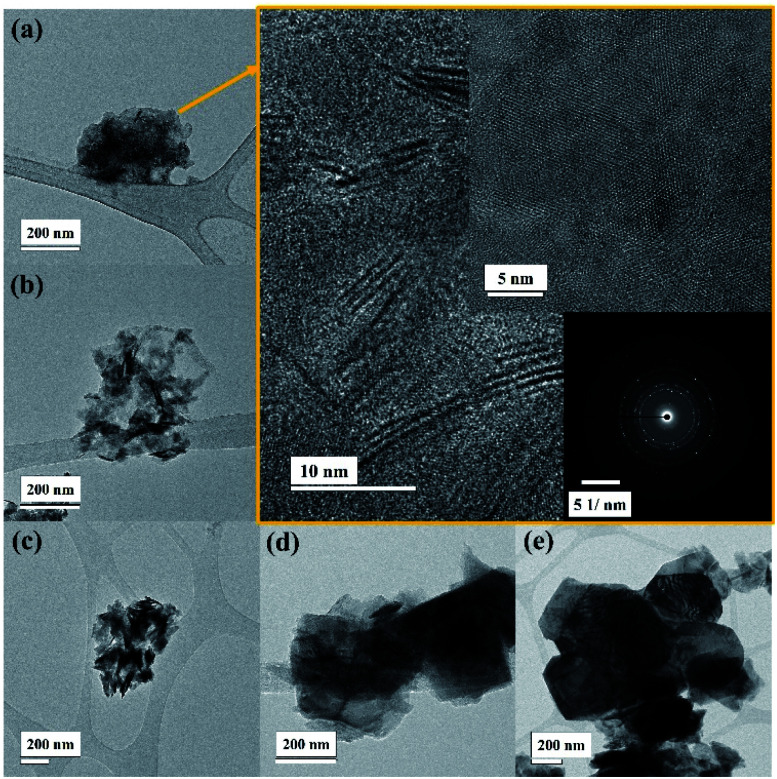
The TEM images showing the size of WSe_2_ flakes at different centrifugal rates (a) ≥12 000 rpm-106 nm, (b) 6000 to 12 000 rpm-255 nm, (c) 3000 to 6000 rpm-295 nm, (d) 1500 to 3000 rpm-395 nm, and (e) ≤1500 rpm-955 nm.

**Fig. 4:**
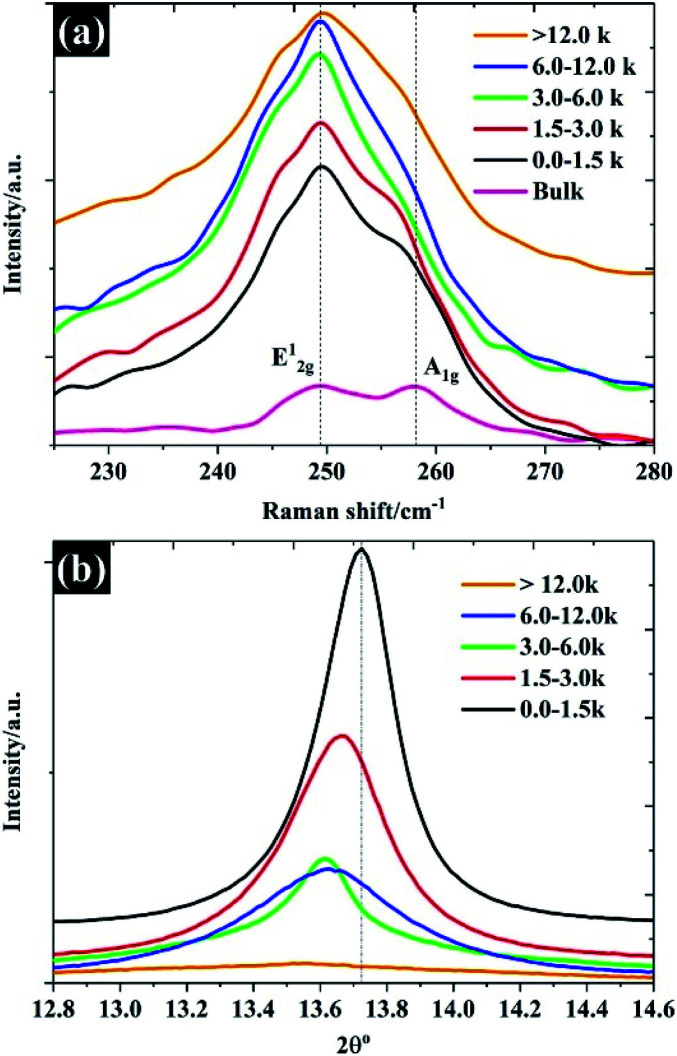
The structural properties of the prepared WSe_2_: (a) Raman spectra and (b) XRD pattern.

The dispersions of WSe_2_ obtained from different centrifugal speeds were characterised using SEM as shown in Fig. 2. The morphology of the exfoliated WSe_2_ exhibits various flake dimensions in terms of both lateral size and flake thickness. At the highest centrifugal rate of over 12 000 rpm, the flake sizes are less than 100 nm (see Fig. 2a). Once the centrifugal speed decreases, the lateral size of the WSe_2_ flakes increases to between 200 nm and 1 μm, as shown in Fig. 2b–e respectively. There is excellent agreement with the lateral size obtained through the DLS analysis, and the results are in accord with previous reports on graphene and MoS_2_ exfoliation.^20–22,28^ Note that the SEM image of the bulk material is shown in Fig. 2f. It is apparent that faster centrifugal speeds diminished not only lateral size but also the flake thickness;^22,28^ and that the dispersions were then further characterised using TEM, Raman, and XRD as shown in Fig. 3 and 4.

The TEM image in Fig. 3 displays a similar flake size where the high centrifugation speed exhibits small flakes as well as low thickness. It is found that the centrifugal speed over 12 000 rpm provides a lateral flake size between 20 nm and 100 nm (see Fig. 3a), confirming the results from DLS and SEM techniques. Moreover, the high resolution TEM images (gold colour box) clearly present mono- to few-layer WSe_2_ nanosheets, and the WSe_2_ atoms are arranged in the 2H phase as can be seen in the inset image. Then again the WSe_2_ flakes become larger and thicker when the centrifugal rate is reduced, which can be seen from Fig. 3b–e, respectively.

The Raman spectra shown in Fig. 4a show predominantly Raman features for each of the flake sizes. The Raman spectra of bulk WSe_2_ show two dominant peaks at approximately 249 cm^−1^ and 257 cm^−1^, which can be assigned to the E^1^_2g_ and A_1g_ modes.^29^ It was found that after the exfoliation and separation processes, the intensity of the A_1g_ peak diminished and shifted to a lower frequency, indicating fewer layers.^30^ For monolayer WSe_2_, there is no frequency difference between E^1^_2g_ and A_1g_ peaks, and the two peaks share the same position.^30^ It is evident that higher centrifugal speeds provided a flake thickness from a single layer to few layers, as the E^1^_2g_ and A_1g_ share the same position. Until the number of layers increases to four layers, there is no significant change in the Raman feature.^31^ To further confirm the thickness of the WSe_2_ flakes, the peak of 2*θ* – about 13.7° of the XRD pattern, referring to the (002) reflection plane of 2H-WSe_2_ – is shown in Fig. 4b. Note that the interlayer spacing of the bulk material is calculated at approximately 0.68 nm. After exfoliation, it can be clearly seen that the FWHM decreases in accordance with the increases of the WSe_2_ flakes representing the diminishing number of layers,^19^ which once again reflects similar results from the SEM, TEM, and Raman techniques. Moreover, it was found that the (002) plane shifted to a lower degree once the flake size became smaller, which reflects the increase of WSe_2_ layer spacing.^32^

**Fig. 5:**
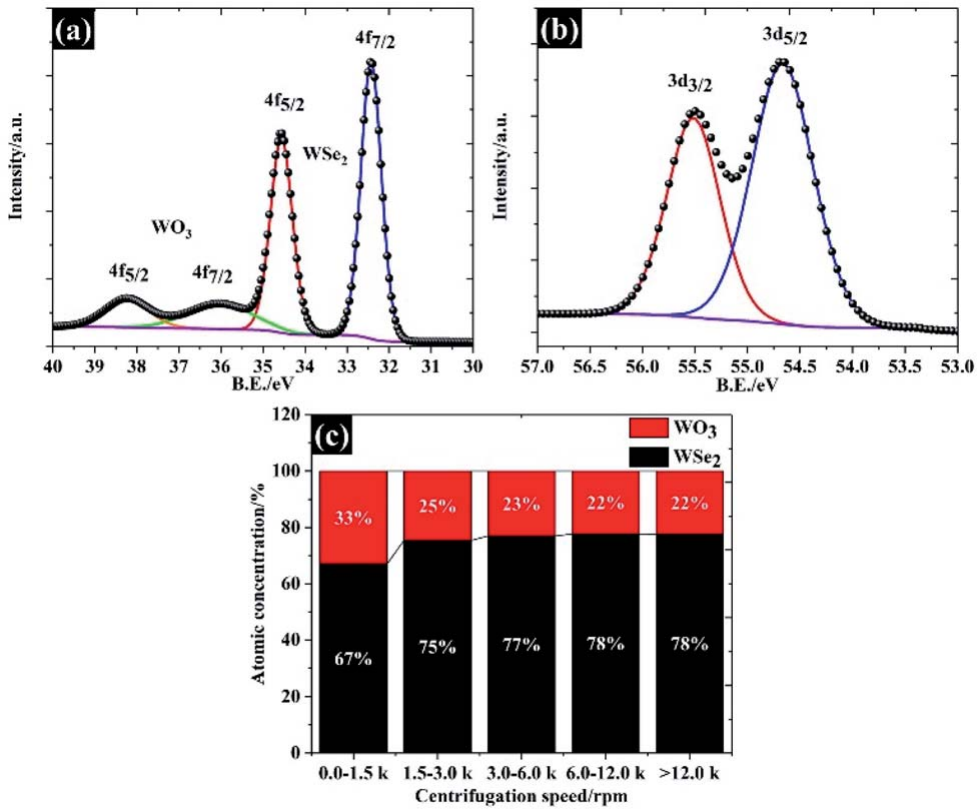
Surface chemical composition of size selected WSe_2_ flakes characterised using the XPS technique: (a) W 4f spectra, (b) Se 3d spectra resulting in an average lateral size of 106 nm, and (c) the atomic concentration between WSe_2_ and WO_3_.

In order to explain the surface chemical composition of the exfoliated materials, the XPS in Fig. 5a and b show the W 4f and Se 3d of 106 nm flakes (quoted as “>12.0k”), respectively. The XPS spectra under other centrifugal conditions are given in Fig. S1 and S2.† Overall, the 106 nm WSe_2_ flakes display four peaks, at 32.4 eV, 34.5 eV, 36.5 eV and 38.3 eV, which can be divided into two components: (1) W 4f_7/2_ and W 4f_5/2_ of W Se_2_, and (2) W 4f_7/2_ and W 4f_5/2_ of the W–O bond, also referring to (W^6+^) of tungsten as shown in Fig. 5a.^33^ This oxide form is likely to have been caused by the partial oxidation at the edge and a defective atom in the WSe_2_. This feature is typically observed in the TMD family.^18^ Apart from the tungsten element, the narrow spectra of Se 3d in Fig. 5b show two peaks at 54.6 eV and 55.4 eV, providing a peak separation of 0.8 eV, which agrees with the binding energy of WSe_2_ crystals.^34^ We further analysed the atomic concentration between WSe_2_ and WO_3_ in each of the flake sizes (see Fig. 5c), and found that the exfoliation process could be used to remove any surface oxide and provide a higher yield of WSe_2_ of up to 78% by atomic concentration for the 106 nm WSe_2_ flakes.

**Fig. 6:**
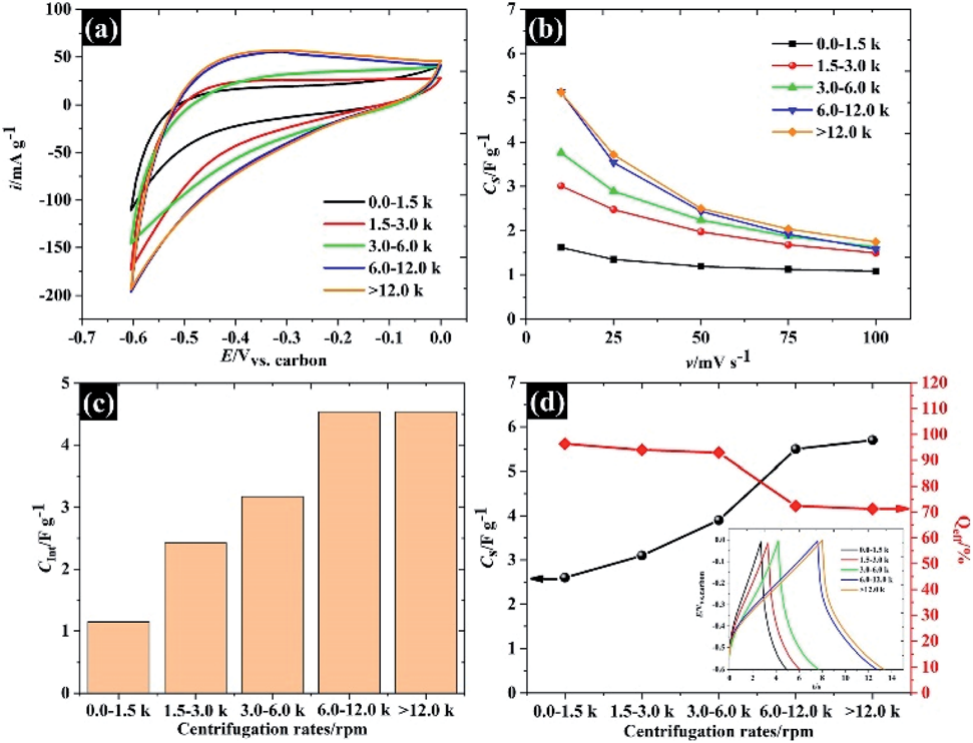
The capacitive properties of the size selected WSe_2_: (a) CV at 10 mV s^−1^, (b) specific capacitance *vs.* scan rates, (c) diffusion controlled capacitance, and (d) specific capacitance and coulombic efficiency, with the inset showing the charge/discharge 0.5 A g^−1^.

To explore the applicability of the WSe_2_ in the electrochemical context further, the electrochemical properties of each of the flake sizes were explored using CV and GCD in 0.5 M KCl as shown in Fig. 6. Overall, it was clear that all the WSe_2_ flake sizes exhibited a small vertex potential window of about 0.6 V (from 0.0 to −0.6 V *vs.* carbon) due to the catalytic properties of WSe_2_ for the hydrogen evolution reaction (HER)^35^ (see Fig. 6a). It was also noted that the CV in Fig. 6b of 106 nm flakes provides the highest specific current. The capacitance of 106 nm flakes is about 5.1 F g^−1^ at 10 mV s^−1^, and decreases to 1.7 F g^−1^ after increasing the scan rate to 100 mV s^−1^. These quoted capacitances are lower than those that have occasionally been reported for the TMD family, this is because there are no conductive additives or polymer binders added. So far, the mass of these additives is often excluded from the calculations.^18^ Meanwhile, it was found that the WSe_2_ flakes provide higher specific capacitance than those of previously reported TMD materials such as MoS_2_ (3.4 F g^−1^), WS_2_ (3.5 F g^−1^), and TiS_2_ (4.6 F g^−1^) under the same measurement conditions and electrode preparation procedures.^32^ This may therefore provide an opportunity for further development of WSe_2_ composite electrodes similar to MoS_2_. Apart from the outstanding electrochemical performance of the smallest WSe_2_ flakes (106 nm), there is a capacitance dependence on associated scan rates for all flake sizes, as can be seen in Fig. 6b. This is due to the limit of ionic diffusion to the WSe_2_ galleries,^19^ and it has been surmised that pseudocapacitive contributions are involved in evaluating the total capacitance. We therefore subsequently analysed the current through the surface and diffusion controlled processes according to eqn (3).^36^

3 *i*(*V*) = *k*_1_*v* + *k*_2_*v*^1/2^

where *k*_1_*v* and *k*_2_*v*^1/2^ correspond to the current from the surface controlled process and the diffusion controlled process, respectively. The total capacitance of WSe_2_ is dominated by the diffusion controlled process, either through surface redox or ion intercalation as shown in Fig. 6c. The charge storage mechanisms of WSe_2_*via* the diffusion controlled process are summarised below.^18,19^

4 (WSe_2_)_surface_ + K^+^ + e^−^ ↔ (WSe_2_^−^K^+^)_surface_

5 WSe_2_ + K^+^ + e^−^ ↔ WSe − SeK

It is evident that the dimensions of smaller flakes provide a significantly higher pseudocapacitance than the dimensions of the larger flakes. One possible reason for this is that we successfully removed the oxide forming at the edge or defect sites of WSe_2_ leading to higher pseudocapacitive contributions from the WSe_2_ component. However, there is also a contribution from the physisorption process as shown in eqn (6).^19^ The diminished capacitance may also be due to the poor surface area of large WSe_2_ flakes.

6 WSe_2_ + K^+^ ↔ WSe − SeK_ads_^+^

Note that the total capacitances, including physisorption (also known as EDLC), surface redox and ion intercalation as well as the coulombic efficiency for each flake size measured using the GCD technique, are given in Fig. 6d. This total capacitance agrees with the capacitance calculated using the CV technique.

To further confirm the charge storage mechanisms of the prepared electrode material, the EIS were then described according to the complex model of capacitance using eqn (7)–(9).^37^

7 *C* = *C*′ − *jC*′′

8
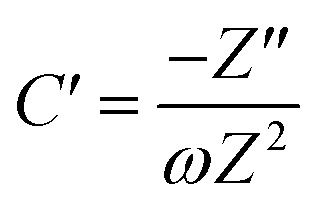
9
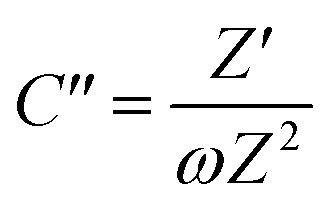


**Fig. 7:**
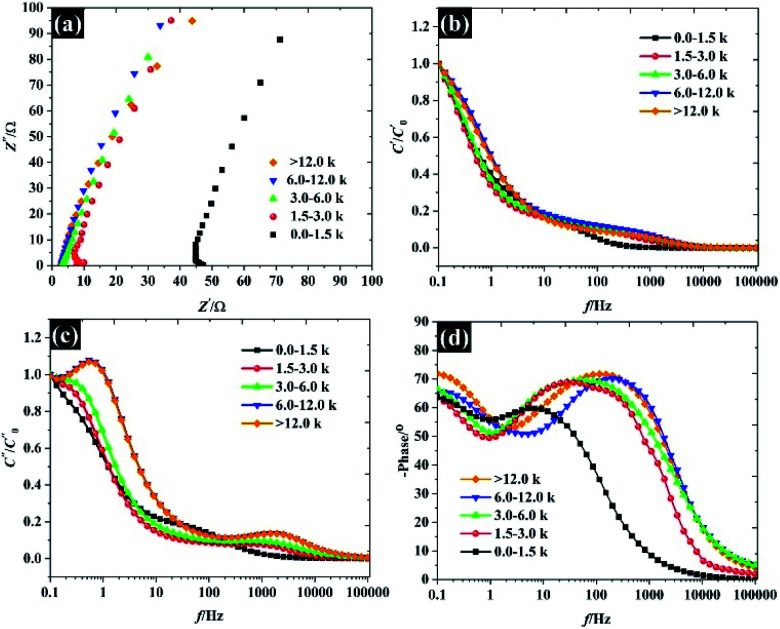
Analysis of the electrochemical impedance spectra of the free-standing size selected WSe_2_ electrode: (a) Nyquist plot, (b) normalised real part capacitance, (c) normalised imaginary part capacitance, and (d) Bode plot.

Overall, the Nyquist plots of all flake sizes in Fig. 7a show a straight line near the vertical axis indicating capacitor behaviour. However, it is noticeable that the internal resistance (*R*_s_) relies on the flake dimensions, with the *R*_s_ becoming larger as the flake dimensions increase. This also explains why the capacitance of larger WSe_2_ flakes is smaller than those of the smallest flakes. Apart from the resistance, the normalised real part capacitance in Fig. 7b refers to the characteristic of the electrode/electrolyte interface.^38^ Evidently, the real part capacitance of all flake sizes decreases as the frequency increases in a similar pattern due to the identical characteristics of the electrode materials and the electrolyte. In contrast, the response across the frequency range from the imaginary part capacitance behaves the other way round for each of the flake sizes (see Fig. 7c). It is noticeable that the peak of about 1 Hz increases with the decrease in flake dimensions. This is due to the increase in the pseudocapacitive contribution (*i.e.* the ion intercalation) which confirms the calculations we arrived at in the previous section. Moreover, the Bode plot in Fig. 7d shows different phase angle responses across the applied frequency, referring to a diverse relaxation time constant value. It was found that the time constant decreased from 167 ms to 4.53 ms as the flake size was reduced.

**Fig. 8:**
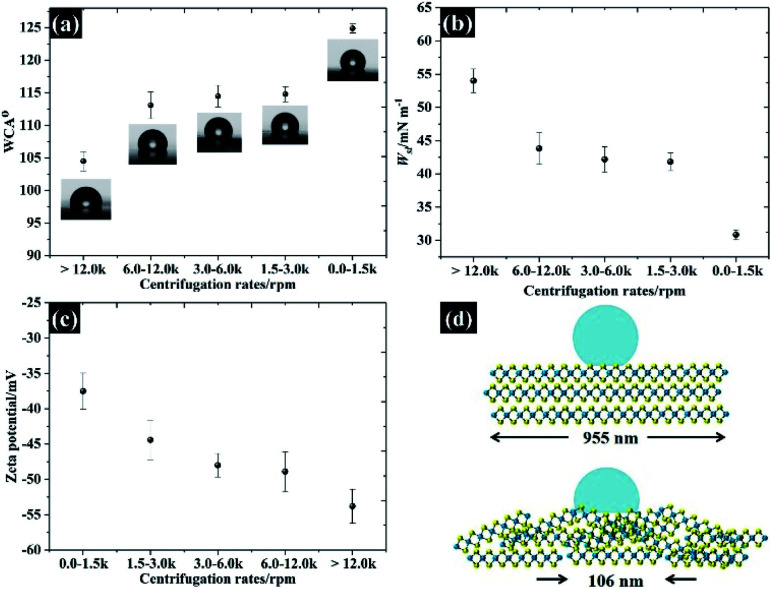
The wettability of free standing WSe_2_ electrodes: (a) the water–air contact angle, (b) the work of adhesion, (c) the zeta potential for each lateral size, and (d) a schematic showing the water–air contact angle of the smallest and largest flake sizes.

Varying the flake size not only changed the electrochemical properties but also changed the wettability of the exfoliated materials as shown in Fig. 8a. It was found that the hydrophobicity of the water increases as the flake sizes grew larger, with the WCA from small to large flakes being roughly 104°, 113°, 114°, 114°, and 124°, respectively. To further explain the interaction between WSe_2_ flakes and water, Young's equation (eqn (2)) was then applied. By assuming the constant *γ*_lv_ for a given liquid–air behaviour (*i.e.* similar water–air tension through all samples) and neglecting the *γ*_sv_, eqn (2) can be rewritten to represent the work of adhesion (*W*_sl_) according to eqn (10):

10 *W*_sl_ = *γ*_lv_(1 + cos *θ*)

where *W*_sl_ is the reversible thermodynamic work required to separate the interface from the equilibrium state between two phases to an infinite distance.^39^ It is clear that the *W*_sl_ in Fig. 8b decreases with increasing flake size, confirming that lower energy is required to separate two phases when the flake size is larger. This suggests that improvements in the electrochemical properties may refer to the wettability of WSe_2_ due to the increase in interfacial contact between the electrolyte and solid interfaces. Meanwhile we know that the electrostatic repulsion is responsible for the wettability,^40^ and we have then calculated the zeta potential for each of the WSe_2_ dispersions as shown in Fig. 8c. Obviously, the WSe_2_ sheets are negatively charged, and the zeta potential then decreases from −37.5 mV to −53.8 mV when the flake sizes become smaller due to the increase in the edge charge population. This means that smaller flakes can provide higher hydrophilic properties as defined by the edge effect (see the schematic in Fig. 8d). This agrees with previous findings on MoS_2_.^41^

**Fig. 9:**
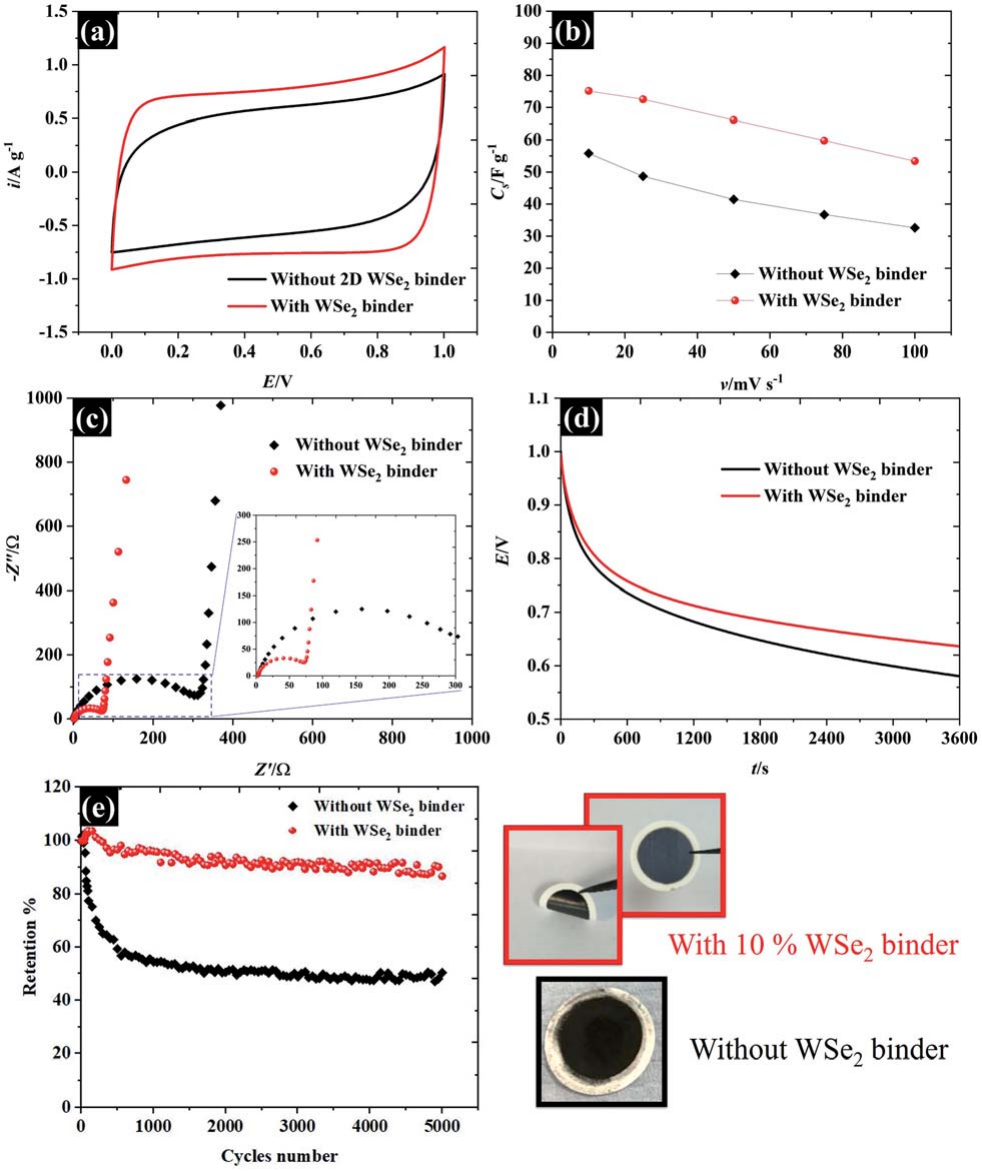
Demonstrating the applicability of the WSe_2_ binder for supercapacitor electrodes in 0.5 M KCl: (a) CV at 10 mV s^−1^, (b) the specific capacitance in respect of the scan rate, (c) EIS at the open circuit potential, (d) the self-discharged behaviour, and (e) cycling stability at a charge/discharge rate of 1 A g^−1^ as well as the images of the prepared electrodes.

The applicability of WSe_2_ nanoflakes in supercapacitors is presented in Fig. 9. The smallest WSe_2_ flakes (quoted as “106 nm flakes”) were chosen as a 2D binder (an additive) in the supercapacitor electrode, instead of using typical conductive additives and polymer binders.^42^ We have introduced new concepts by using exfoliated TMD materials as pseudocapacitive binders for an alternative option for high performance supercapacitor electrodes (either typical ones or flexible/free-standing electrodes). Overall, we added 10% WSe_2_ when preparing an activated carbon electrode. The WSe_2_ flakes provide van der Waals interaction, through which they can bind with the carbon particles. The CV in Fig. 9a shows the success of using WSe_2_ as a 2D binder. It is evident that the CV of the activated carbon with WSe_2_ binder shows a more rectangular shape than the activated carbon without the WSe_2_ binder, indicating higher capacitive properties and improved electrical conductivity.^43^ After adding the WSe_2_ binder, the capacitance increased by about 35 percent at 10 mV s^−1^ (from 55.8 F g^−1^ to 75.1 F g^−1^). It was noted that the rate capability of an activated carbon supercapacitor (without adding WSe_2_) decreased by 70% after increasing the scan rate to 100 mV s^−1^, while the activated carbon electrode (with WSe_2_) decreased by only 40% (see Fig. 9b). This improvement is higher than the results achieved in previous reports, reduced graphene oxide as a 2D conductive binder,^7^ and far better than the results obtained using the MXene film binder.^44^ For comparison, the supercapacitor performances of activated carbon with PVDF are presented in Fig. S5.† The supercapacitor performance was then investigated using the EIS technique as shown in Fig. 9c. It can be clearly seen that results for both the “with WSe_2_ binder” and the “without WSe_2_ binder” provide a resistance solution (*R*_s_) of 0.3 Ω due to the similar electrolytes used. However, the electrode without the WSe_2_ binder presents a much greater semi-circle as can be seen in Fig. 9c, suggesting that the WSe_2_ binder could reduce charge transfer resistance (*R*_ct_) at the electrode interfaces, which is in accord with the CV result. Note that the *R*_ct_ of the activated carbon with the WSe_2_ binder, and the sample without the WSe_2_ binder is about 73 Ω and 312 Ω respectively. In the lower frequency regions, the Nyquist plot displays a straight line close to the vertical axis, suggesting ideal capacitor properties.^38^ Moreover, it is clear that after adding the WSe_2_ binder, the self-discharging behaviour of the supercapacitor improved by about 9% as shown in Fig. 9d, where the potentials after a 1 h test are about 0.64 V (with WSe_2_) and 0.58 V (without WSe_2_). As a final point, we successfully used WSe_2_ nanoflakes as a pseudocapacitive binder, and the WSe_2_ can bind the activated carbon particles together as shown in Fig. 9e. The supercapacitor with 10% WSe_2_ binder shows excellent cycling stability up to ∼90% retention while the pure AC supercapacitor displays only 50% retention after 5000 cycles. Moreover, it is clear that an electrode with WSe_2_ provides a smooth and flexible surface, which blends without cracking. Meanwhile, an electrode without WSe_2_ displays a rough surface of agglomerate-activated carbon particles. These results show a great potential for using WSe_2_ nanoflakes as an electrode binder as well as an excellent potential for the preparation of composite materials in the future development of energy storage technologies.

## Conclusions

In summary, the WSe_2_ nanoflakes were produced *via* liquid phase exfoliation in a water/*n*-propanol solution. The flake sizes were finely selected using the centrifugal method, which produced a variety of flake sizes from about 100 nm to 1000 nm. The electrochemical properties of each flake size were explored, and it was found that the smallest flakes (average flake size of 106 nm) provided the best electrochemical performance due to their excellent pseudocapacitive properties as well as their wettability. Furthermore, we have demonstrated the applicability of using WSe_2_ as an electrode binder, replacing typical PVDF or PTFE binders. Once WSe_2_ is applied, it improves the overall performance of the supercapacitors, increasing the capacitance by about 35% (from 55.8 F g^−1^ to 75 F g^−1^), reducing the charge transfer resistance by about 76 percent, and improving the self-discharge by approximately 9 percent. This work introduced an alternative process for preparing an energy storage electrode, which should lead to the continuing development of energy storage applications.

## Conflicts of interest

There are no conflicts of interest to declare.

## Supplementary Material

NA-003-D0NA00592D-s001
