# Supplementary material for: Controlling the flake size of bifunctional 2D WSe2 nanosheets as flexible binders and supercapacitor materials
Source: Nanoscale Adv. 2020 Sep 30;3(3):653–60. doi: 10.1039/d0na00592d (PMC9418638; doi:10.1039/d0na00592d)
Supplement: NA-003-D0NA00592D-s001 [file NA-003-D0NA00592D-s001.pdf]

Electronic Supplementary Material (ESI) for Nanoscale Advances.
This journal is © The Royal Society of Chemistry 2020

Supplementary Data

Controlling the Flake Size of Bifunctional 2D WSe_2 Nanosheets as Flexible Binders and Supercapacitor Materials

Pawin Iamprasertkun[a, b, *], Wisit Hirunpinyopas[c], Varisara Deerattrakul[d], Montree Sawangphruk[b], Chakrit Nualchimplee[a]

[a]Department of Physics, Faculty of Sciences and Liberal Arts, Rajamangala University of Technology Isan, Nakhon Ratchasima, 30000, Thailand.

[b]Department of Chemical and Biomolecular Engineering, School of Energy Science and Engineering, and Centre of Excellence for Energy Storage Technology (CEST), Vidyasirimedhi Institute of Science and Technology, Rayong, 21210, Thailand

[c]Department of Chemistry, Faculty of Science, Kasetsart University, Bangkok 10900, Thailand.

[d]Department of Chemical Engineering, Faculty of Engineering, Kasetsart University, Bangkok 10900, Thailand.

Keyword: WSe_2, liquid phase exfoliation, Flakes Size, Binder, Supercapacitor

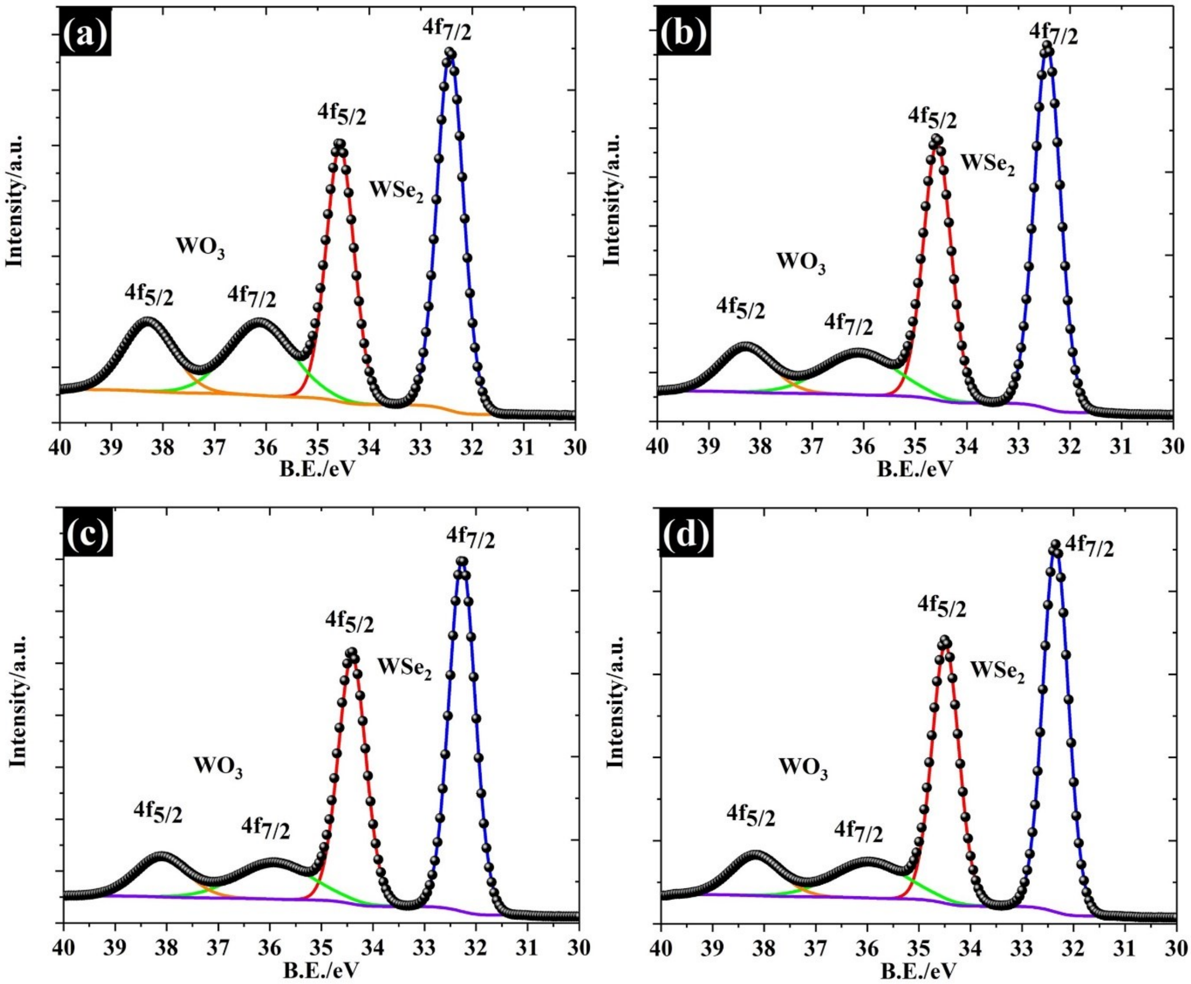

Fig. S1. The W4f XPS spectra of the different centrifugation speeds (a) 0.0-1.5 k, (b) 1.5- 3.0 k, (c) 3.0-6.0 k, and (d) 6.0-12.0 k.

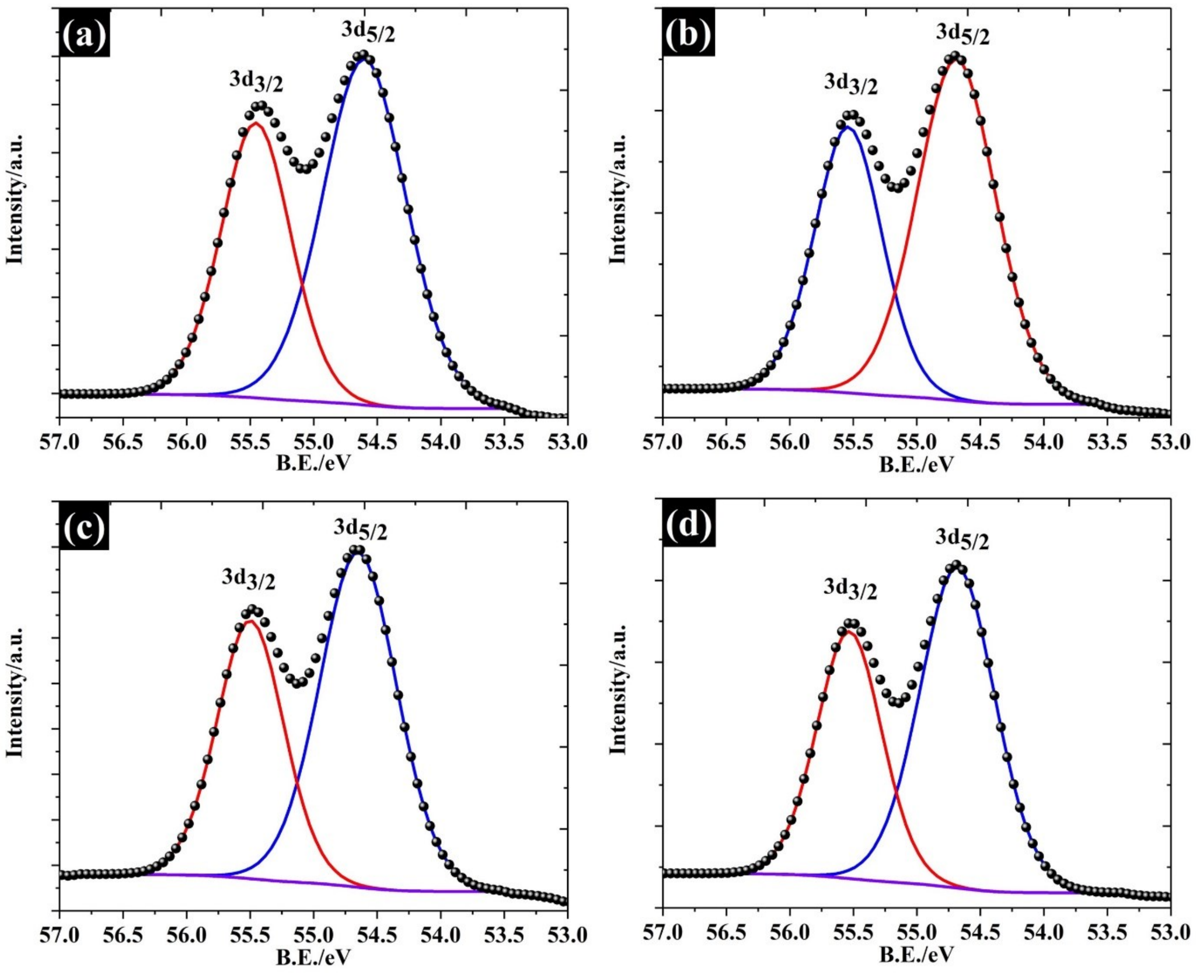

Fig. S2. The Se3d XPS spectra of the different centrifugation speeds (a) 0.0-1.5 k, (b) 1.5- 3.0 k, (c) 3.0-6.0 k, and (d) 6.0-12.0 k.

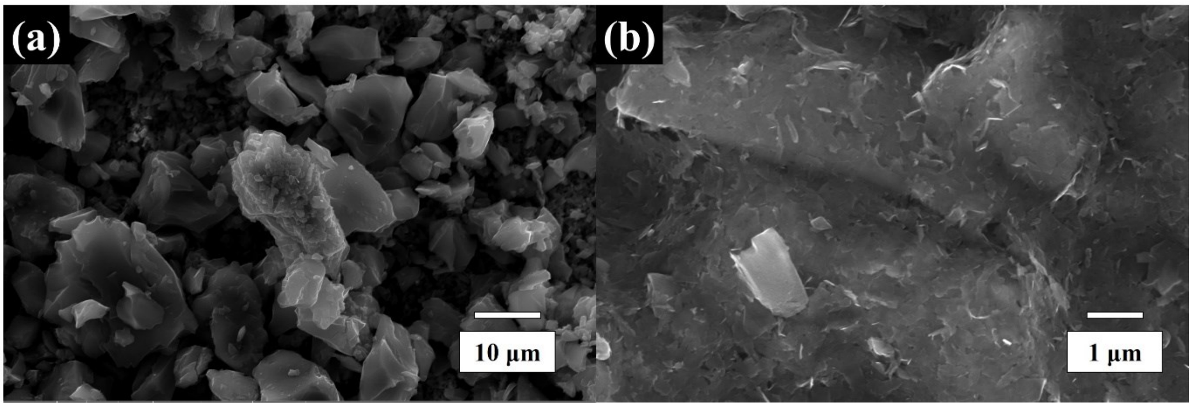

Fig. S3: the SEM images of the prepared electrodes (a) activated carbon without WSe_2 binder, and (b) activated carbon with WSe_2 binder.

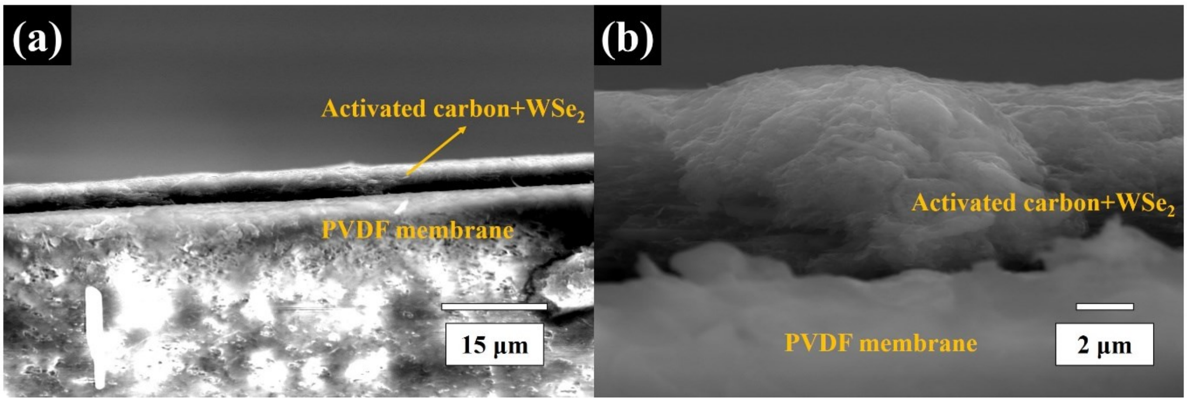

Fig. S4: the cross-sectional SEM images of the prepared activated carbon with WSe_2 binder (a) low magnification, and (b) high magnification.

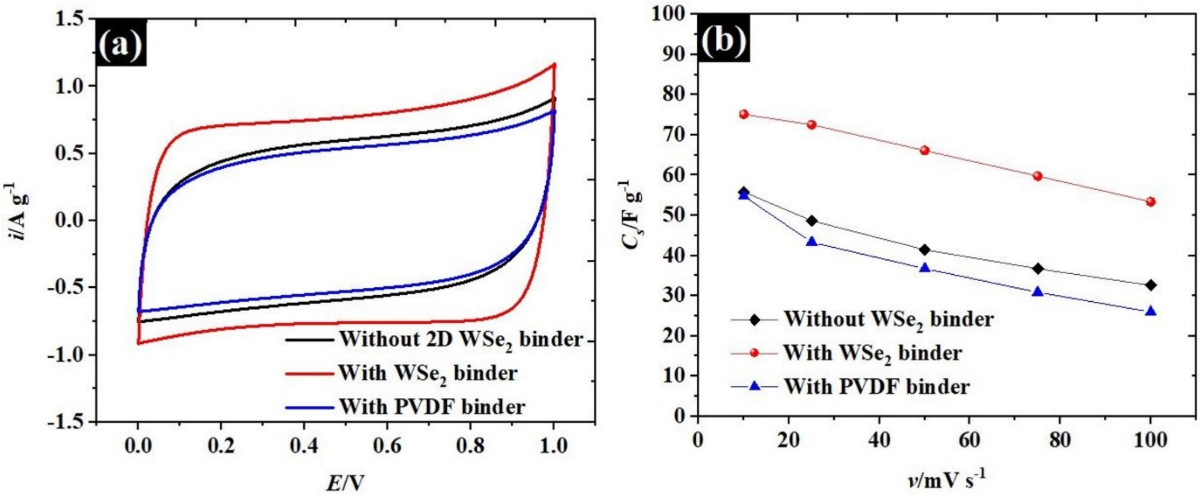

Fig. S5: the electrochemical performance of the as-prepared activated carbon electrode with 10 % of PVDF as a binder compared with the candidate electrodes.

Table 1. The electrical conductivity of WSe_2 and prepared electrodes.

Samples	R/Ω	A/cm^2	L/µm	σ/S m^{-1}
WSe_2 (106 nm)	2.88	0.762	2.48*	0.01126
WSe_2 (255 nm)	3.01	0.762	2.48*	0.01081
WSe_2 (295 nm)	3.02	0.762	2.48*	0.01074
WSe_2 (396 nm)	8.44	0.762	2.48*	0.00385
WSe_2 (955 nm)	47.2	0.762	2.48*	0.00068
AC+WSe_2	2.69	0.762	6.42**	0.03129

*the thickness of the electrode were obtain from the linear function between thickness and mass of TMDs materials (see ACS Nano 2017, 11, 11, 11082–11090)
**the thickness of the electrode was obtain from cross-sectional SEM image in Fig. S4
